# Thoughts about “other” patients’ rights during COVID-19 pandemic

**DOI:** 10.18502/jmehm.v13i11.4386

**Published:** 2020-09-16

**Authors:** Dimosthenis Chrysikos, Constantinos G. Zografos, George C. Zografos

**Affiliations:** 1 *Consultant Surgeon, First Department of Propaedeutic Surgery, Hippocration Hospital, School of Medicine, University of Athens, Athens, Greece.*; 2 *Surgeon, First Department of Propaedeutic Surgery, Hippocration Hospital, School of Medicine, University of Athens, Athens, Greece.*; 3 *Professor of* *Surgeon, First Department of Propaedeutic Surgery, Hippocration Hospital, School of Medicine, University of Athens, Athens, Greece.*

**To the Editor**

 Pandemic, (originates from Greek word παν, pan, that means 'all' and δήμος, demos, which refers to people') is a global outbreak in most cases, of an infectious disease, that has spread over multiple countries or continents (1). According to World Health Organisation (WHO), “ischemic heart disease, stroke, chronic obstructive lung disease and lower respiratory infections have been estimated as the leading causes of death worldwide during the past decade “(2). For a disease or condition to be a pandemic, merely besides being widespread or lethal, it should also be infectious or contagious. On that basis, cancer, trauma and other chronic diseases that cause increasing numbers of deaths worldwide, are not considered a pandemic because these diseases are neither infectious nor contagious.

**Past pandemics and lessons to learn**

In history, there have been a number of severe pandemics. One of the most catastrophic pandemics recorded in human history was the Bubonic plague (The Black Death) caused by a bacterium known as Yersinia pestis. This pandemic has been reported to have killed around 75–200 million people in the 14th century, through Asia, Africa and Europe. Another devastating pandemic that wiped out millions of people was the Spanish flu that took place in 1918 and more recently the H1N1 flu pandemic that occurred in 2009 (3). 

Nowadays, pandemics include HIV/Acquired Immune Deficiency Syndrome and the 2019 Coronavirus disease, which was declared by the WHO a pandemic on 11 March 2020. 

Past pandemics in human history have as a consequence to gain experiences from ancient cultures by the way the reacted to contagious diseases. Even in Middle Ages, ancient doctors of that period have observed that there might be a correlation between time exposure and the outbreak of symptoms of lethal and contagious diseases like the Black Death. It has also been noted that, after a period of observation, patients who had not developed symptoms of the illness would potentially not be affected and remarkably, would not have the capability to spread the disease upon entering the ancient city (4). To that point, mandatory isolation was the first measure taken historically. Nowadays, this form of social distancing is called “quarantine”. 

The scientific and medical community during outbreaks of infectious diseases has to adopt measures regarding prevention, immunization and antimicrobial treatments, in order to preserve the good of public health. Through this process there are many lessons to learn and practices to avoid in the future. 

Historically, it has been proved that from the Spanish influenza pandemic of 1918, virtually all deaths were due not to influenza itself, but to complicating secondary infections. The lack of vaccination and pharmaceutical modalities targeting the influenza virus, has led the medical community and the authorities to adopt measurements that would potentially restrict the worldwide scale spread of the viral disease. Such measurements were control efforts to isolate the people, social distancing by abandoning public gatherings, quarantines and optimal personal hygiene by every means (5,6).

Despite the catastrophic effects of the Spanish influenza on the global civilization, the defence was to fade it from the public and scientific attention. One of the possible explanations towards this attitude, might be the fact that the pandemic was overshadowed by more fundamental historical events, such as World War I (6). 

**The ongoing COVID-19 pandemic**

Certainly, the new pandemic of COVID-19, is an emerging, rapidly evolving situation. Governments worldwide without exception, have focused to fight the pandemic and support measures recommended by the WHO. In order to defeat the pandemic, all current financial resources are redistributed to provide prevention, detection, treatment and recovery of COVID-19 disease. For instance, Intensive Care Units are to be tremendously expanded and medication or new vaccines need to be developed. This is the most serious global challenge.

Nowadays, medical and political systems and particularly the politicians face a global criticism, if their response to pandemic fails to effectively restrain it. 

We should all admit that during the pandemic, the medical profile, in the most of the countries, has not changed at all. Mortality and morbidity rates due to the COVID-19 will occupy an unknown ratio that most probably will not make us reconsider the top ten causes of death worldwide (2). This raises the question: What about “other” patient’s rights-that have not been infected by the corona virus disease?

**Human rights and patients’ rights**

The Universal Declaration of Human Rights is considered a milestone effort to record in a document the human rights. It was created in 1948 in Paris (7) and continues to inspire the concept of the person, the fundamental dignity and the equality of all human beings. 

Patients' rights vary all over the world, due to various ethics, cultural and social forms. It is a fact that the fundamental patient-physician relationship reflects the relationship of citizens with the state and the authorities (8).

It has been shown that during the pandemic, screening examinations such as mammography and colonoscopy are postponed. Undoubtedly, this is attributed not only due to personal responsibility, but due to the fear that has been evolved by mass media along with state health measurements like quarantines and social distancing. To postpone medical visits, or screening examinations might mean that existed or forthcoming diseases might change their course or stage and could even deteriorate in the future. 

In addition, many potentially vulnerable general practitioners or older medical doctors with multiple comorbidities who belong to high risk groups, avoid examining their own patients with symptoms suspecting the pandemic disease, in fear of dissemination of the viral disease. As a result, they prefer to refer them to the dedicated public hospitals. The problem that emerges is that chronic or forthcoming diseases are underestimated or overlooked. This situation endangers patients’ rights to health care providers. 

Access to outpatient’s departments is also restricted, according to the measurements that have been taken. The number of patients with cardiovascular disease is significantly decreased, this is mainly due to the fact that patients postpone their visit to the hospitals. However, mortality and morbidity rates of cardiovascular diseases have remained stable (2).

**The oncological patients**

Patients with malignant neoplasms should continue to fight and undergo specialized treatment without feeling that they are 'depriving' the lives of other people who are suffering and succumbing to a pandemic currently affecting the global community. On the contrary, they are doubly anxious at this stage of dealing with such difficult diseases while at the same time being aware that they have an increased risk of mortality from COVID-19 infection.

For instance, patients with hematologic malignancies and solid tumors are at high risk for serious complications of the disease due to the immunosuppression they cause, both the neoplastic disease itself and the treatments given to treat it. It is therefore necessary to systematically apply to this vulnerable group of the population, measures to prevent the transmission and spread of the corona virus.

In the field of ​​therapeutic interventions (chemo, radiotherapy) there are recommendations from ASCO (American Society of Clinical Oncologists) and other related organizations (9).

Regarding patients who have completed treatment and are on follow-up, it is recommended to postpone their reassessment visits in order to avoid unnecessary burden on health structures of preventive measures and transmission of the virus. Patients also undergoing maintenance treatment with intravenous therapy, may be postponed or given longer intervals, depending on their disease and the phase of treatment.

On the contrary for those patients who have active disease, either at an early stage, locally advanced, or metastatic, and need special treatment, it should not be missed or postponed. Other patients with abdominal symptoms or other non-lethal symptoms should not be disregarded. In other cases, all other symptoms are attributed to the pandemic. This may lead to erroneous differential diagnosis.

Corresponding phenomena could be observed by the delay in chemotherapy or radiotherapy and transplantation. It is therefore important to evaluate each case individually, so that these patients are not deprived of necessary interventions at the right time, but also that the health system's resources are not wasted unnecessarily. It is equally important for all of us to take and comply with all necessary measures to reduce the pandemic, so that health and social structures can meet the needs of all other patients suffering from other diseases, and especially those most vulnerable to infections (9).

**A vicious cycle of panic during the COVID-19 pandemic**

Another, equal important parameter is patients’ phobias to come to hospital. It is surprising to see how many people do not attend in the premises of the hospital, in the fear of contacting with COVID-19. In case patients seek medical help during the pandemic, they have to wait for many hours to be examined and after having been tested negative for COVID-19, leading to delayed presentations for several sometimes even fatal cases. 

Medical doctors from their side, should provide informed and written consent about the risk to operate or perform an invasive examination procedure to non-symptomatic patients during the pandemic. For example, delaying oncological surgeries could lead to disease progression and to tumors that are no longer surgically excluded, limiting patients' chance of healing. Moreover, patients have the right to life-saving interventions and this responsibility lies in medical systems and governments that fight pandemics. 

Another thought provoking aspect that should be addressed during the COVID-19 outbreak, is medical doctors’ professional independency. Physicians, who follow up other patients during the COVID-19 pandemic in the majority of developed countries, are not in the position to decide on the allocation of medical resources. As a consequence, the most appropriate treatment must be provided to their patient at all times. It prevails the medical approach of “one size fits at all” that means personalized medicine is disregarded. Indeed, resource allocation is the responsibility of policymakers and health care providers while defining the right policies might be very helpful in respecting the other patients’ rights during the COVID-19 pandemic.

Everyone has the right to health without discrimination. Patients belong to a certain group of population. Patients with disabilities, older patients and those living in extreme poverty, as well as patients in detention, the homeless, refugees and other minority communities need special government support.

Prevention and increased care by physicians and nurses and in general from those who provide medical support it is crucial to this difficult time for mankind. At this phase patient need apart from increased medical care, understanding, vigilance, and psychological support.

It sounds simple but it is not. The mass of population has to be convinced that the wide acceptance of the restrictive measures during pandemics is critical. These interventions might be criticized that they violate human rights and are against the principles of democracy. To get the balance right, back to normal life and everyday activities to the best extent possible, it is mandatory, when the pandemic fades away (10). 

## Conclusions

In conclusion, the ongoing COVID-19 pandemic has led governments and healthcare professionals worldwide, to adopt mandatory restrictive measurements. The scientific and medical community have focused by every means to fight the COVID-19 pandemic. As a result, the oncological patients and other patients with chronic diseases who have not been infected are at risk, because their medical visits are postponed or because of fear of infection, they are not followed up from their medical doctors. This situation endangers those patient's rights who belong to subgroup of vulnerable population. 

Societies and governments have to respond effectively to the global threat of COVID-19 pandemic, however, we must not let it overshadow everything and overlook the needs, the personalized therapy and follow up of the non-infected patients who have the right to continue to have access to the services of the public health system. 

Finally, it is of paramount importance to learn from our history. We cannot afford to repeat past mistakes from previous devastating pandemics, by neglecting other patients, when battling the inevitable pandemic of COVID-19 or other pandemics in the future.

## Notes:

**Citation to this article:**

Chrysikos D, Zografos CG, Zografos GC. Thoughts about “other” patients’ rights during covid-19 pandemic. J Med Ethics Hist Med. 2020; 13: 11.

## References

[1] Porta M (2008). Dictionary of Epidemiology.

[2] Anonymous Health statistics and information systems.

[3] Dumar AM (2009). Swine Flu: What You Need to Know.

[4] Sehdev PS (2002). The origin of quarantine. Clin Infect Dis.

[5] Garcia-Sastre A, Whitley RJ (2006). Lessons learned from reconstructing the 1918 influenza pandemic. J Infect Dis.

[6] Parmet WE, Rothstein MA (2018). The 1918 influenza pandemic: lessons learned and not-introduction to the special section. Am J Public Health.

[7] Anonymous United Nations: Universal Declaration of Human Rights.

[8] Anonymous WHO: Patients’ rights.

[9] Gosain R, Abdou Y, Singh A, Rana N, Puzanov I, Ernstoff MS (2020). COVID-19 and cancer: a comprehensive review. Curr Oncol Rep.

[10] Mehta A, Quinn TC (2016). Addressing future epidemics: historical human rights lessons from the AIDS pandemic. Pathog Immun.

